# The National Dementia Workforce Study: The Plan for Organization Sample Frames and Data Collection

**DOI:** 10.1111/jgs.70036

**Published:** 2025-09-04

**Authors:** James Wagner, Laura M. Wagner, Sheryl Zimmerman, Johanna van Tyen Silbersack Hickey, Kate Stewart, Sandi Nelson, Ji Qi, Raphael Nishimura, Piotr Dworak, Margaret Hudson, Jennifer Kelley, Heidi Guyer, Amy R. Pettit, Donovan T. Maust, Joanne Spetz

**Affiliations:** ^1^ Survey Research Center, University of Michigan Ann Arbor Michigan USA; ^2^ Department of Community Health Systems, School of Nursing; Philip R. Lee Institute for Health Policy Studies; and Healthforce Center at UCSF University of California San Francisco California USA; ^3^ School of Social Work, University of North Carolina at Chapel Hill Chapel Hill North Carolina USA; ^4^ Cecil G. Sheps Center for Health Services Research, University of North Carolina at Chapel Hill Chapel Hill North Carolina USA; ^5^ Mathematica, Inc Princeton New Jersey USA; ^6^ DLH Durham North Carolina USA; ^7^ RTI International Durham North Carolina USA; ^8^ Independent Consultant Boston Massachusetts USA; ^9^ Department of Psychiatry University of Michigan Ann Arbor Michigan USA; ^10^ Center for Clinical Management Research, VA Ann Arbor Healthcare System Ann Arbor Michigan USA; ^11^ Philip R. Lee Institute for Health Policy Studies and Healthforce Center at UCSF University of California San Francisco California USA

**Keywords:** data collection, dementia, establishment survey, sampling, workforce

## Abstract

The National Dementia Workforce Study was designed to improve our understanding of the individuals and systems who care for people with dementia, but designing and implementing such a study is challenging due to the large number of patient care organizations, clinical and direct care roles, and locations in which care is provided. Specifically, developing a probability sample of organizations and staff caring for people with dementia is a complex and difficult process. While there are national sampling frames available for federally certified nursing homes (i.e., via data from the Center for Medicare and Medicaid Services), there are no national sampling frames for assisted living communities or home care agencies. The latter frames must be developed through querying state‐level regulatory agencies and through other, supplemental strategies such as working with professional organizations, large employers, and organizations that provide services (e.g., payroll services) to this sector. Further, since there are no national sampling frames that allow for direct sampling of staff working in any of these types of organizations, we opted for a two‐stage design. In the first stage, organizations are identified, sampled, recruited to participate in an organizational‐level survey, and asked to provide a roster of eligible staff. In the second stage, individual staff members are recruited for a staff‐level survey. We describe the plan for sampling and recruitment procedures to be used in each stage and discuss limitations, including implications for coverage of the target population. Data collected through these surveys will be available to the research community.

**Summary**

- Key points
  ○ Drawing a sample of staff across the long‐term care workforce of people who care for people with dementia is complex; due to their distribution across a wide variety of organizational setting types and locations, different parts of the workforce require different sampling strategies.
  ○ A combination of federal and state resources, including both regulatory data and information collected through industry‐related nonprofits, are needed to gain insight into potential sampling frames.
  ○ The most efficient approach to reaching staff in some components of the workforce is via cluster sampling that recruits nursing home facilities, assisted living communities, and home care agencies that employ these staff.
- Why does this paper matter?
  ○ Creating efficient sampling and recruitment strategies for studies of the workforce caring for people with dementia is a critical step in conducting research on this important population.

## 1 Introduction

During the past several decades, major public investments in data infrastructure—including large, longitudinal, nationally representative surveys that have the capacity to be linked to other data sources—have played an instrumental role in advancing our understanding of the health care needs of the older U.S. population [1, 2, 3]. Survey research has also been key for understanding the clinical workforce that cares for older adults, offering insights into factors associated with the supply of care providers and variation in care delivery and care quality. As the aging population includes a growing number of people with dementia [4], there is an urgent need to improve our understanding of the diverse professional workforce caring for them and to generate data‐based insights into opportunities to improve consistency and quality of care across the care continuum, including community‐based, in‐home, assisted living, and nursing home settings.

Prior studies in these settings have included the biennial National Study of Long‐Term Care Providers (NSLTCP), which began in 2012 and was designed to include adult day services centers, assisted living and similar residential care communities, home health agencies, hospices, and nursing homes [5]. In 2020, the NSLTCP was modified to include inpatient rehabilitation facilities and long‐term care hospitals and was renamed the National Post‐acute and Long‐term Care Study (NPALS) [6]. It generates facility‐level data on the supply and use of services, supplemented by administrative data and by questions about sampled facility residents or service users. It does not focus on individual clinicians or staff. To date, efforts to sample individual workforce members have primarily focused on single professions or settings. These have included the National Nursing Home Survey, last conducted in 2004 [7], and the National Home and Hospice Care Survey [8]. The latter, last conducted in 2007, included a companion effort (the National Home Health Aide Survey) that used separate procedures and asked a sample of home health aides to complete a questionnaire [9]. These efforts produced important findings on training [10], the type of care provided [11, 12], workplace injuries [13], and factors driving turnover [14], underscoring the value of workforce studies. A pilot study of long‐term care direct care workers was included in the 2024 wave of the NPALS [6], but overall, there is a dearth of recent workforce information across the continuum of care and we lack the detailed information on the knowledge, experiences, characteristics, and employment outcomes of individual workforce members that is necessary to assess and optimize their ability to care for people with dementia.

Research on the professional dementia care workforce in the U.S. has been limited in part due to the complexity of sampling and recruiting relevant organizations and the staff who work in them. As a result, many studies to date have sampled organizations but have not recruited staff or have relied on administrative data (e.g., the Payroll Based Journal) to capture information. Whereas setting‐specific investigations are necessary and valuable, they are unable to capture additional dynamic and interconnected components of the workforce; for example, multiple professionals work within single settings, and single occupations (e.g., nurses) are represented in multiple settings. In recognition of these gaps and the resources that would be required to address them, in 2022 the National Institute on Aging (NIA) released a Request for Applications focused on characterizing the dementia care workforce. The resulting National Dementia Workforce Study (NDWS) is designed to be both the first coordinated, nationally representative family of surveys to span the continuum of care (creating a stand‐alone source of comprehensive workforce data obtained directly from the workforce itself) and to be linked to numerous other available datasets (e.g., administrative and claims data) to generate insights into the relationships between workforce issues and clinical outcomes. As with other major NIA‐funded data infrastructure initiatives, the resulting data will be made available to the research community.

In addition to a Community Clinician Survey, the NDWS focuses on three key practice settings in which people with dementia receive care—nursing homes, assisted living communities, and home care agencies—and on the largest occupations within those settings, including licensed nurses (registered nurses and licensed practical/vocational nurses), nursing assistants, personal care aides, and activity staff. Our efforts to define the sample frame for each of these three settings and to sample individuals from them are described here. The Community Clinician Survey and the development of survey questionnaires for nursing homes, assisted living, and home care are described elsewhere in this special collection.

## 2 Methods

### 2.1 Overview of Sampling Approach and Goals

Our overarching goal was to obtain a nationally representative sample of care organizations and direct care staff caring for people with dementia in three key settings: nursing homes, assisted living communities, and home care. We sought to construct the sample frame for each setting to be nationally representative of the components of the dementia care workforce in that setting (e.g., representing all licensed nurses working in assisted living).

In developing our sampling approaches, we convened a multidisciplinary team of experts with relevant methodological expertise and with content expertise specific to the regulations, licensure, routine practices, and workforce within each setting. Whenever possible, we drew on methods used successfully in prior national studies. The team also drew on prior experience developing, fielding, and analyzing a variety of surveys of health care professionals [15, 16, 17, 18].

We used a two‐stage design. In the first stage, we sought to identify and recruit a sample of organizations (nursing homes, assisted living communities, and home care agencies, respectively) to enable implementation of an organization‐level survey and allow us to request access to individual staff. We stratified organization lists using variables such as measures of size, rurality, proportion of residents or population in the local area who pay for health care via Medicaid, and region in order to reduce sampling error. Stratifying by organization size also helps to control the size of the staff sample. In the future, these strata can also be used to oversample organizations that provide care to underserved populations. In the second stage, we sought to sample and recruit individual staff to complete a survey. In addition, because the NDWS surveys are designed to be longitudinal in order to assess workforce changes over time, we incorporated this perspective into our sampling approach.

### 2.2 Stage 1: Organizational Sample Design

#### 2.2.1 Nursing Homes

To construct a sampling frame of nursing homes caring for people with dementia, we used the most recent data available from three primary sources, each of which contained unique necessary data elements. The first source, the Minimum Data Set (MDS) for 2020 and 2021, contains federally mandated, individual‐level assessments of all nursing home residents and is available from the Centers for Medicare and Medicaid Services (CMS). In all CMS‐certified nursing homes, MDS assessments must be completed within 14 days of admission, at least quarterly thereafter, and at discharge; thus, MDS data can be used to calculate census numbers for individual facilities. Our second source was 2021 Long‐Term Care: Facts on Care in the U.S. (known as LTCFocus) facility‐level data, which allowed us to determine whether nursing homes had a specialty care unit for dementia and the percentage of residents whose primary support was Medicaid. Our third source was January 2024 Nursing Home Compare, a publicly available CMS dataset that contains facilities' names, addresses, characteristics such as bed size and ownership, and quality indicators. This allowed us to determine whether facilities identified in our first two data sources were still operating in 2024 and to measure the number of certified beds in each facility and in rural locations. We linked the three sources using a common data element, the unique CMS Certification Number, or CCN, to construct our sample frame of certified U.S. facilities identified in the 2021 MDS that were still operating in January 2024.

To calculate each facility's average daily census in 2021, of both all residents and of residents with dementia (using items I4200_ALZHMR_CD and I4800_DMT_CD [19]), we used 2021 MDS data. We also used 2020 data to identify stays in 2021 that started in the prior year. The facility‐level file at the end of this step contained *N* = 15,498 facilities that submitted any MDS assessments in 2021. We merged the 2021 LTCFocus (*N* = 15,050 facilities) and the January 2024 Nursing Home Compare (*N* = 14,906 facilities) with the MDS facility‐level file, using CCN. The 2021 LTCFocus had fewer facilities than the 2021 MDS file because it did not include nursing homes in Alaska or the District of Columbia. The 2024 Nursing Home Compare file had fewer facilities than either of the two other files due to more facility closures than openings over time. We retained all facilities that had observations in the 2021 MDS and 2024 Nursing Home Compare files (*N* = 14,816). We dropped duplicate records (*n* = 2) and facilities located in U.S. territories (*n* = 6), for a final total of *N* = 14,808 facilities in the sample frame. We also geo‐coded facility addresses from Nursing Home Compare to determine whether the facility was in a rural census tract, based on Rural–Urban Commuting Area (RUCA) codes [20].

We used several characteristics available on the sampling frame for stratification, including the number of certified beds as a proxy measure of facility size (categorized as low, medium, or high based on tertiles of the number of certified beds: 1–80, 81–120, 121+), the proportion of residents paying via Medicaid (split into “low” and “high” based on the median of 64%), and rurality (with rural defined as RUCA codes 7–10). Given the relatively small number of nursing homes in rural areas, we collapsed strata by the number of certified beds (medium and high) and within those strata, collapsed the proportion of residents paying via Medicaid (low and high) in order to create a separate stratum (stratum 1). Rural and Medicaid populations are both underserved, and these strata can be used for oversampling in the future. Table 1 shows the resulting strata and population and sample counts by stratum.

**TABLE 1:** Population and sample counts of nursing homes by stratum.

Stratum	Number of certified beds	Proportion paid by Medicaid	Rurality	Population count	Population percent	Sample count	Sample percent
1	Medium and High	Low and High	Rural	1073	7.2%	20	10.0%
2	Low	Low	Not Rural	2338	15.8%	6	3.0%
3	Low	Low	Rural	861	5.8%	10	5.0%
4	Low	High	Not Rural	1317	8.9%	6	3.0%
5	Low	High	Rural	612	4.1%	32	16.0%
6	Medium	Low	Not Rural	2261	15.3%	30	15.0%
7	Medium	High	Not Rural	2295	15.5%	38	19.0%
8	High	Low	Not Rural	1701	11.5%	44	22.0%
9	High	High	Not Rural	2350	15.9%	14	7.0%
Total				14,808	100%	200	100%

*Note*: Categories for number of certified beds are: 1–80 (low), 81–120 (medium), and 121+ (high). Medicaid proportion is categorized as low or high based on the median of 64%. Rural indicates Rural–Urban Community Area (RUCA) code of 7–10.

Sampling rates across the strata were set proportional to an estimate of the staff size in each stratum. We estimated staff size based on total hours reported in the Payroll Based Journal (PBJ), a CMS‐developed system for mandated reporting of facility staffing [21]. We focused on eligible categories of staff (expressed as hours per resident) and the number of residents. The goal was to produce an equal probability sample of staff across all strata with roughly equal cluster sizes.

#### 2.2.2 Assisted Living Communities

In contrast to nursing homes, which are cataloged at the national level due to CMS regulations, the vast majority of care delivered in assisted living settings is not reimbursed by Medicare or Medicaid; thus, these settings are not easily visible in claims data. Although assisted living communities are regulated at the state level, the licensure terms used to define “assisted living” vary from state to state, and the accessibility of each state's list of licensed communities also varies. As a result, constructing a national frame requires defining “assisted living” in each state, interacting with the websites of the various regulatory agencies, and downloading relevant data or calling state regulators to request data.

For Year 1 (Wave 1, starting in 2024) of the NDWS's Assisted Living Survey, we focused on a subsample of states. We sorted a list of the 50 states and the District of Columbia by their total population of individuals aged 65 years and older, estimated from the American Community Survey, an annual demographics survey conducted by the Census Bureau. From the sorted list of states, we used systematic sampling with a random start to assign states to replicates. This created four replicates of 10 states each and one replicate with 11 states. Each of these replicates is a random subsample of all states. To obtain our Wave 1 subsample, we randomly selected two of the replicates (resulting in inclusion of 21 states) and constructed assisted living community lists for them. In Wave 2, we will expand the frame to include the remaining three replicates of states and updated lists for the two replicates included in Wave 1.

As with the nursing home sample, we began by drawing on existing data sources. The National Center for Assisted Living (NCAL), which is part of the American Health Care Association (AHCA), leads national advocacy, education, networking, professional development, and quality initiatives on behalf of assisted living providers. NCAL tracks the various licensure terms used across the U.S. for “assisted living.” We started by searching the websites of state‐level regulatory agencies for the relevant licensure terms. (We will repeat this process in future year sampling efforts, to allow for the fact that additional licensure terms may emerge.) When the results were not available for download, we contacted officials of the regulating agency and requested the list.

We defined a minimum set of variables that would be needed from each state, including contact information for the agencies (name, address) and a measure to assess the size of the assisted living community (number of licensed beds). In cases where the number of beds was not available (*n* = 157, about 1%), we imputed the median value. We added several variables from other sources, including the Census Region and the RUCA code. We also excluded assisted living communities with fewer than four licensed beds, following the eligibility definition employed by the National Post‐acute and Long‐term Care Study.

In the next step, we created strata based upon the number of licensed beds, the Census Region, and rural status. We divided the bed sizes into four groups (4–6, 7–34, 35–99, and 100+). These groupings were initially based on tertiles (4–6, 7–34, and 35+), but we decided to break out 100+ as a category because communities of that size are regulated differently in some states. We also found that the Northeast Census Region had too few assisted living communities to be included separately, and we collapsed it with the Midwest Region. Given that rural assisted living communities make up about 6% of all communities, we opted to treat rural location as a separate stratum. We allocated sampled assisted living communities to each stratum proportionate to the number of licensed beds in the stratum. Table 2 shows the stratification variables and population and sample counts by stratum.

**TABLE 2:** Count of assisted living communities in population and sample by stratum.

Stratum	Bed size category	Census Region/rurality	Population count	Sample count
1	4–6	Midwest‐Northeast	1215	4
2		South	1456	4
3		West	6417	12
4	7–34	Midwest‐Northeast	669	4
5		South	1058	4
6		West	864	4
7	35–99	Midwest‐Northeast	1383	26
8		South	1372	26
9		West	643	12
10	100+	Midwest‐Northeast	801	32
11		South	788	32
12		West	690	32
13	All	Rural (all regions)	762	8
Total			18,118	200

*Note*: Midwest and Northeast regions were combined due to small population size in the Northeast region. Due to low prevalence of rurality (defined as a Rural–Urban Community Area [RUCA] code of 7–10), all rural assisted living communities were grouped together in their own stratum, regardless of region.

#### 2.2.3 Home Care Agencies

There is no national registry of home care agencies available. In constructing our sampling frame approach, we considered that two types of agencies provide home‐based services: home health agencies, which typically focus on short‐term services reimbursed by Medicare and register with CMS, and home care agencies, which typically focus on long‐term support for people living with disabilities (including dementia) and register with state regulatory agencies. Some agencies provide both types of services. Previous surveys have focused on home health agencies [22], but people with dementia often need long‐term support, and thus we sought to develop a sample frame that includes the latter group.

We undertook a comparison of the two sets of agencies (those registered with CMS and those registered with state regulatory agencies) in several states. We found that in one of the five replicates (which included Florida, Indiana, Louisiana, Minnesota, Nevada, New Hampshire, Ohio, Vermont, Virginia, and West Virginia), about 10% of all agencies were only on the CMS‐registered list, 6% were on both the CMS‐registered list and the lists obtained from the states, and 84% were only on the lists obtained from the states. We took into account that there were likely to be ineligible units (e.g., agencies that provide home care for children) on the state lists, which may have inflated the actual number and percentage of agencies that appear only on the state lists.

Given time constraints, we opted to start by sampling from the list of home health agencies that are registered with CMS. We excluded agencies located outside of the 50 states and DC and any that did not report offering “Home Health Aide Services” (*n* = 832). This left us with 11,023 eligible agencies. In Year 2 (Wave 2), we will develop a sampling frame based upon state‐level lists of home care agencies.

Available CMS data include several variables relevant for sampling. We used the number of episodes of Medicare payment as a proxy for agency size. We also used ZIP code data to attach information from the American Community Survey about the population in the ZIP Code Tabulation Area (ZCTA) of the home health agency and to identify the RUCA code. We also added Census Region and the percentage of persons whose insurance is Medicaid alone (from the American Community Survey). From these, we chose Census Region, rural status, the number of episodes of payment by Medicare, and an indicator variable for whether the proportion of the ZCTA population of the home health agency that is insured by Medicaid only is greater than the median (13.5%). As was the case with assisted living communities, the Northeast Region had a small proportion of the home health agencies (8.5%) and we collapsed it with the Midwest Region. Given the small number of home health agencies in rural areas, we treated them as a separate stratum (in keeping with the approach in the assisted living sample) and only separated agencies in rural areas for further stratification using the binary classification of the proportion of the population that has “Medicaid only” for insurance, to create two separate rural strata. (As noted previously, those who rely on Medicaid for insurance and those who live in rural areas are underserved populations.) For the number of episodes of payment by Medicare, we split the agencies into two groups using the median value (393) as a cutoff value. This resulted in the 14 strata shown in Table 3.

**TABLE 3:** Stratification of Home Health Agencies for Sampling.

Stratum	Rural	Number of episodes group	Census Region/rurality	Proportion Medicaid only in ZCTA > 13.5%	Population count	Sample size
1	Not Rural	Low	Midwest‐Northeast	Below	677	12
2		Low	Midwest‐Northeast	Above	514	10
3		High	Midwest‐Northeast	Below	1049	18
4		High	Midwest‐Northeast	Above	907	16
5		Low	South	Below	863	16
6		Low	South	Above	630	12
7		High	South	Below	1374	24
8		High	South	Above	968	18
9		Low	West	Below	551	10
10		Low	West	Above	838	16
11		High	West	Below	858	16
12		High	West	Above	1139	20
13	Rural	Both	All	Below	231	4
14				Above	424	8
Total					11,023	200

*Note*: Midwest and Northeast regions were combined due to small population size in the Northeast region. The number of episodes is two groupings created by splitting cases at the median number of episodes of payment from Medicare (393). Proportion Medicaid only in ZCTA divides the frame into two groups – those above and below the median (13.5%). Due to low prevalence of rurality (defined as a Rural–Urban Community Area [RUCA] code of 7–10), rural assisted living communities were stratified by the percentage of the population paying via Medicaid variable and created two rural strata.

We allocated sampled home health agencies to each stratum proportionately to the number of agencies in the stratum. We made sure that each stratum had a minimum of four sampled agencies allocated to it. We then rounded allocations to integer values.

### 2.3 Stage 2: Staff Sample Design

Stage 2 of our sampling approach involves obtaining lists of all eligible staff from participating organizations, a process known as “rostering,” and then sampling staff from these lists. This approach was employed by the National Home Health Aide Survey [9] and the National Nursing Assistant Survey [23]. A key benefit of this approach (as opposed to obtaining lists from licensing entities or other sources) is that it enables us to identify both licensed and unlicensed professionals who provide care in these settings. To identify staff in our roles of interest, the study team developed a list of eligible titles for each staff survey (see Table S1), which were provided to organizations that participated in the administrator survey. The staff lists provided as part of rostering were then mapped onto these eligible titles. Part‐time and contract staff were considered to be eligible. The sampling rates were set within each stratum such that we expected an approximately equal probability sample of staff. One exception is staff with a “Director of Nursing” or analogous title, who were selected with certainty.

## 3 Data Collection

NDWS survey implementation and data collection protocols and timelines for the nursing home, assisted living, and home care surveys were designed to occur in two stages, paralleling the sampling design. In the first stage, facilities, organizations, and agencies are recruited. An administrator is invited to complete a short survey and is asked to provide a staff roster. In the second stage, a sample of staff from the roster is selected and invited to complete a survey.

For Wave 1 (fielded from August 2024 through early March 2025), we conducted outreach to 200 entities (e.g., individual facilities or agencies) for each setting category, with the goal of recruiting 100 entities for each of the three surveys for the first survey wave. At the facility or organization level, participation entailed a facility designee completing an administrator survey and submitting a staff roster. Then, as described above, the NDWS team selected a sample of staff from each of the rosters. Our goal was to have 1100 completed surveys from nursing home staff (i.e., 11 staff from each of 100 respondent organizations), 900 completed surveys from staff working in assisted living communities (i.e., nine staff from each of 100 respondent organizations), and 800 completed surveys from staff working for home health agencies (i.e., eight staff from each of 100 respondent organizations). Among organizations that agreed to participate, we sought to achieve an 80% response rate among sampled staff.

The sampled entities in each survey received mailed prenotification letters, mailed invitation letters, email invitations, and telephone calls prompting them to participate. We offered an incentive both to the entity and to the administrator responsible for completing the questionnaire and submitting the roster. The administrator survey takes about 22 min to complete.

Sampled staff were invited to participate in a 25‐minute web‐based survey. Invitations were sent directly to staff via a mailed packet, which included a recruitment letter with information about the study, the survey website, and log‐in credentials. Follow‐up communications included email invitations and a series of reminder emails, letters, and postcards. An incentive was offered for completing the questionnaire. The survey also requested contact information from all respondents to facilitate annual follow‐up surveys, which will allow us to follow staff respondents who remain in the same position as well as those who have left their positions for new employment within health care or who have transitioned to different industries.

Additional detail about survey implementation methods and response rates will be the subject of a later publication.

### 3.1 Accounting for the Sample Design in Analysis

As is typically true for large national surveys, data analysis will need to account for features of the sampling design. For each of the three NDWS surveys described here, analysts will need to account for the stratification of the organizations [24]. This applies to both data collected about the organization itself (i.e., the administrator survey) and data collected from the staff selected from each organization. Analysis of the staff surveys will need to account for the clustering of the staff within agencies. The NDWS team will calculate and provide sample weights to account for differing probabilities of selection, adjustments for nonresponse, and post‐stratification to known population totals and will provide resources for data users to facilitate accurate analyses.

## 4 Discussion

The methods described here represent a major step forward in efforts to understand the dementia care workforce in the U.S. Obtaining a comprehensive picture of this workforce requires efforts to engage workers operating in a variety of settings and organizational structures. Nursing home sample frame development is aided by the availability of national data on federally certified facilities. Building sampling frames for surveys of assisted living communities and home care agencies is more challenging, especially given variation between U.S. states. For all sample frames, however, careful planning is required to identify the relevant sampling units and strata. For each of the sample frames described here, we stratified sampling using variables that reflect rurality and Medicaid‐related use because people living in rural areas and those with low income, as proxied by Medicaid‐related use, are often underserved [25, 26, 27].

As described here, the NDWS surveys employ a two‐stage sample design approach, selecting organizations in the first stage and sampling staff from recruited organizations in the second stage. This panel design is both efficient for data collection and a powerful tool for analysts working to understand this workforce. Together with organization‐level data gathered through administrator surveys (e.g., policies, practices, size), this approach aids assessment of “inner setting” and organizational factors affecting care delivery, which is critical to understanding both current practice and efforts to modify practice. Further, each survey will be conducted annually, enabling longitudinal analysis, and the sample design will allow researchers to answer questions about the current workforce caring for people with dementia and to analyze the dynamic changes to that workforce over time. This will support innovative research on career trajectories and turnover. Specifically, our future plans include drawing new samples of the workforce in each setting (nursing homes, assisted living communities, and home care agencies). This will allow us to identify new entrants into the workforce while also conducting follow‐up surveys with staff who responded in prior survey waves. Follow‐up will allow us to identify those who exit their jobs as well as those who exit the dementia workforce altogether, while monitoring those who remain. In sum, we will have a complete picture of the dynamics of this workforce, including stable members, new entrants, and those who exit.

At each of these stages of sampling, there are recruitment tasks. The first stage involves recruitment of organizations. This involves finding a person with the authority to make a decision about participation and finding someone willing to complete the survey on behalf of the facility and supply a roster of staff. The second stage aims to recruit staff from the responding organizations. Our survey implementation approach considered barriers to recruitment and added features to try to reduce those barriers. For example, while staff members are under no obligation to participate in the survey, we offer an incentive to compensate them for their time and hopefully to increase their willingness and motivation to participate. We also use multiple contacts across different modes (e.g., email, phone).

There are some limitations that need to be mentioned. We described some of the limitations with respect to coverage of the home care agencies. In future survey waves, we expect to supplement current sampling frames to improve coverage. We also know that some assisted living communities will not appear on our frame due to our requirement that there be at least four certified beds. We could also miss new assisted living communities and others that are not yet regulated by the state in which they are located. In addition to improving coverage of home care agencies, in the future, we also expect to detect new nursing homes, assisted living communities, and home care and home health agencies. We are interested in changes in the population. Therefore, identifying new organizations, that is, “births,” will be important.

While the NDWS surveys will be an invaluable stand‐alone data source, the NDWS infrastructure will allow respondent information to be linked to actual care and outcomes experienced by people with dementia as derived from Medicare and Medicaid claims or MDS assessment data. This will afford the remarkable opportunity to examine how workforce characteristics are associated with actual care delivery and outcomes experienced by people with dementia across the country. Prior studies have employed similar linkages to advance our understanding of many health care issues, such as the association between the death of a spouse and subsequent health care utilization [2], and the relationship between nurse workforce characteristics and patient outcomes in hospitals. We encourage researchers who are interested in exploring NDWS data to visit the study website at www.ndws.org.

## Author Contributions

Concept and design: All authors. Acquisition, analysis, or interpretation of data: Not applicable. Drafting of the manuscript or revising critically for important intellectual contributions: All authors. Final approval of the published version: All authors.

## Conflicts of Interest

The authors declare no conflicts of interest.

## Linked Articles

This publication is part of the special collection of National Dementia Workforce Study. To view all articles under this special collection visit https://agsjournals.onlinelibrary.wiley.com/doi/toc/10.1111/(ISSN)1532‐5415.national‐workforce‐study.

## Supporting information


**Data S1:** jgs70036‐sup‐0001‐Supinfo.pdf.

## References

[1] J. J. Manly , R. N. Jones , K. M. Langa , et al., “Estimating the Prevalence of Dementia and Mild Cognitive Impairment in the US: The 2016 Health and Retirement Study Harmonized Cognitive Assessment Protocol Project,” JAMA Neurology 79, no. 12 (2022): 1242–1249. 36279130 10.1001/jamaneurol.2022.3543 PMC9593315

[2] L. Lei , E. C. Norton , J. Strominger , and D. T. Maust , “Impact of Spousal Death on Healthcare Costs and Use Among Medicare Beneficiaries: NHATS 2011–2017,” Journal of General Internal Medicine 37, no. 10 (2022): 2514–2520. 35083650 10.1007/s11606-021-07339-7 PMC9360304

[3] J. M. Reckrey , R. S. Morrison , K. Boerner , et al., “Living in the Community With Dementia: Who Receives Paid Care?,” Journal of the American Geriatrics Society 68, no. 1 (2020): 186–191. 31696511 10.1111/jgs.16215 PMC6957088

[4] K. B. Rajan , J. Weuve , L. L. Barnes , E. A. McAninch , R. S. Wilson , and D. A. Evans , “Population Estimate of People With Clinical Alzheimer's Disease and Mild Cognitive Impairment in the United States (2020–2060),” Alzheimer's and Dementia 17, no. 12 (2021): 1966–1975. 10.1002/alz.12362 PMC9013315 34043283

[5] National Center for Health Statistics , “National Study of Long‐Term Care Providers: Survey Methodology and Documentation,” (2013), https://archive.cdc.gov/www_cdc_gov/nchs/data/nsltcp/NSLTCP_survey_methodology_and_documentation.pdf.

[6] National Center for Health Statistics , “National Post‐Acute and Long‐Term Care Study,” accessed March 30, 2025, https://www.cdc.gov/nchs/npals/index.html.

[7] A. L. Jones , L. L. Dwyer , A. R. Bercovitz , and G. W. Strahan , “The National Nursing Home Survey: Overview,” Vital Health Statistics Series 13, no. 167 (2009): 1–155. 19655659

[8] L. L. Dwyer , L. D. Harris‐Kojetin , L. Branden , and I. M. Shimizu , “Redesign and Operation of the National Home and Hospice Care Survey,” Vital Health Statistics Series 1, no. 53 (2010): 1–192. 20737836

[9] A. Bercovitz , A. J. Moss , M. Sengupta , et al., “Design and Operation of the National Home Health Aide Survey: 2007–2008,” Vital Health Statistics Series 1, no. 49 (2010): 1–94. 20648796

[10] M. Sengupta , F. K. Ejaz , and L. D. Harris‐Kojetin , “Training of Home Health Aides and Nurse Aides: Findings From National Data,” Gerontology and Geriatrics Education 33, no. 4 (2012): 383–401. 23095222 10.1080/02701960.2012.702167

[11] A. Bercovitz , M. Sengupta , A. Jones , and L. D. Harris‐Kohetin , “Complementary and Alternative Therapies in Hospice: the National Home and Hospice Care Survey: United States, 2007,” National Health Statistics Reports 33 (2011): 1–20. 25585442

[12] L. L. Dwyer , B. Han , D. A. Woodwell , and E. A. Rechtsteiner , “Polypharmacy in Nursing Home Residents in the United States: Results of the 2004 National Nursing Home Survey,” American Journal of Geriatric Pharmacotherapy 8, no. 1 (2010): 63–72. 20226393 10.1016/j.amjopharm.2010.01.001

[13] D. McCaughey , G. McGhan , J. Kim , D. Brannon , H. Leroy , and R. Jablonski , “Workforce Implications of Injury Among Home Health Workers: Evidence From the National Home Health Aide Survey,” Gerontologist 52, no. 4 (2012): 493–505. 22217463 10.1093/geront/gnr133

[14] R. Stone , J. Wilhelm , C. E. Bishop , N. S. Bryant , L. Hermer , and M. R. Squillace , “Predictors of Intent to Leave the Job Among Home Health Workers: Analysis of the National Home Health Aide Survey,” Gerontologist 57, no. 5 (2017): 890–899. 27106825 10.1093/geront/gnw075

[15] L. Chu and J. Spetz , California Board of Registered Nursing: 2022 Survey of Registered Nurses, (Philip R. Lee Institute for Health Policy Studies, 2024).

[16] L. Chu and J. Spetz , Survey of Health Care Employers in Arizona: Home Health Agencies, 2015 (Healthforce Center at UCSF, 2016).

[17] L. Chu and J. Spetz , Survey of Health Care Employers in Arizona: Long‐Term Care Facilities, 2015 (Healthforce Center at UCSF, 2016).

[18] L. M. Wagner , B. L. Brush , J. B. Engberg , N. G. Castle , and E. Capezuti , “Quality Care Outcomes in Nursing Homes: The Effects of a Nurse's Country of Origin and Education,” Journal of Nursing Regulation 5, no. 4 (2015): 49–56.

[19] J. D. Niznik , J. L. Lund , L. C. Hanson , et al., “A Comparison of Dementia Diagnoses and Cognitive Function Measures in Medicare Claims and the Minimum Data Set,” Journal of the American Geriatrics Society 72, no. 8 (2024): 2381–2390. 38814274 10.1111/jgs.19019 PMC11323171

[20] USDA ERS , “Rural‐Urban Commuting Area Codes,” accessed March 30, 2025, https://www.ers.usda.gov/data‐products/rural‐urban‐commuting‐area‐codes/documentation/.

[21] Center for Medicare and Medicaid Services , “Staffing Data Submission Payroll Based Journal (PBJ),” accessed March 30, 2025, https://www.cms.gov/medicare/quality/nursing‐home‐improvement/staffing‐data‐submission.

[22] A. Bercovitz , A. J. Moss , M. Sengupta , et al., “An Overview of Home Health Aides: United States, 2007,” National Health Statistics Reports 34 (2011): 1–32. 21688727

[23] M. R. Squillace , R. E. Remsburg , L. D. Harris‐Kojetin , A. Bercovitz , E. Rosenoff , and B. Han , “The National Nursing Assistant Survey: Improving the Evidence Base for Policy Initiatives to Strengthen the Certified Nursing Assistant Workforce,” Gerontologist 49, no. 2 (2009): 185–197. 19363014 10.1093/geront/gnp024

[24] S. Heeringa , B. T. West , and P. A. Berglund , Applied Survey Data Analysis (Chapman and Hall/CRC Press, 2017).

[25] E. A. Kramarow , “Diagnosed Dementia in Adults Age 65 and Older: United States, 2022,” National Health Statistics Reports 203 (2024): 1–9. 38912900

[26] M. Kramer , M. Cutty , S. Knox , A. V. Alekseyenko , and A. Mollalo , “Rural–Urban Disparities of Alzheimer's Disease and Related Dementias: A Scoping Review,” Alzheimer's & Dementia: Translational Research & Clinical Interventions 11, no. 1 (2025): e70047. 39935615 10.1002/trc2.70047 PMC11811960

[27] M. Rahman , E. M. White , C. Mills , K. S. Thomas , and E. Jutkowitz , “Rural‐Urban Differences in Diagnostic Incidence and Prevalence of Alzheimer's Disease and Related Dementias,” Alzheimer's & Dementia 17, no. 7 (2021): 1213–1230. 10.1002/alz.12285 PMC8277695 33663019

