# Supplementary material for: The National Dementia Workforce Study: The Plan for Organization Sample Frames and Data Collection
Source: J Am Geriatr Soc. 2025 Sep 4;73(11):3336–43. doi: 10.1111/jgs.70036 (PMC12412904; doi:10.1111/jgs.70036)
Supplement: Supplementary file 1 — Data S1: jgs70036‐sup‐0001‐Supinfo.pdf. [file JGS-73-3336-s001.pdf]

Supplemental Table S1. Eligible Staff Titles, by Setting

**Nursing Homes***	**Assisted Living Communities**	**Home Care Agencies**
7 Registered Nurse	Registered Nurse	Registered Nurse
9 LPN/LVN	Licensed Practical/Vocational Nurse	Licensed Practical/Vocational Nurse
10 CNA	Certified Nurse Aide/Assistant	Certified Nurse Aide/Assistant
11 Nurse Aide in Training	Nurse Aide/Assistant	Nurse Aide/Assistant
12 Medication Aide/Technician	Home Health Aide/Home Health Assistant	Home Health Aide/Home Health Assistant
	Personal Care Aide/Assistant	Personal Care Aide/Assistant
	Activity Staff	Activity Staff

*Numbers for Nursing Home titles indicate the numeric Job Title code from the PBJ Policy Manual.
